# P-1670. SARS-CoV-2 Infection Incidence in Immunosuppressed Persons and Association with Wastewater Variant Circulation

**DOI:** 10.1093/ofid/ofaf695.1844

**Published:** 2026-01-11

**Authors:** Woudase Gallo, Jiashu Xue, Maggie Chahoud, Xori Green, Moreno Rodrigues, Andrew H Karaba, William Werbel

**Affiliations:** Johns Hopkins, Baltimore, Maryland; Johns Hopkins University, Baltimore, Maryland; Johns Hopkins University, Baltimore, Maryland; Johns Hopkins University, Baltimore, Maryland; Johns Hopkins, Baltimore, Maryland; Johns Hopkins University, Baltimore, Maryland; Johns Hopkins University, Baltimore, Maryland

## Abstract

**Background:**

Immunocompromised patients remain at risk for COVID-19 despite multiple vaccinations owing in part to SARS-CoV-2 variant evolution. Infection incidence in the contemporary variant climate and relation to population-level viral circulation are not known, which impairs clinical guidance.

**Figure:**
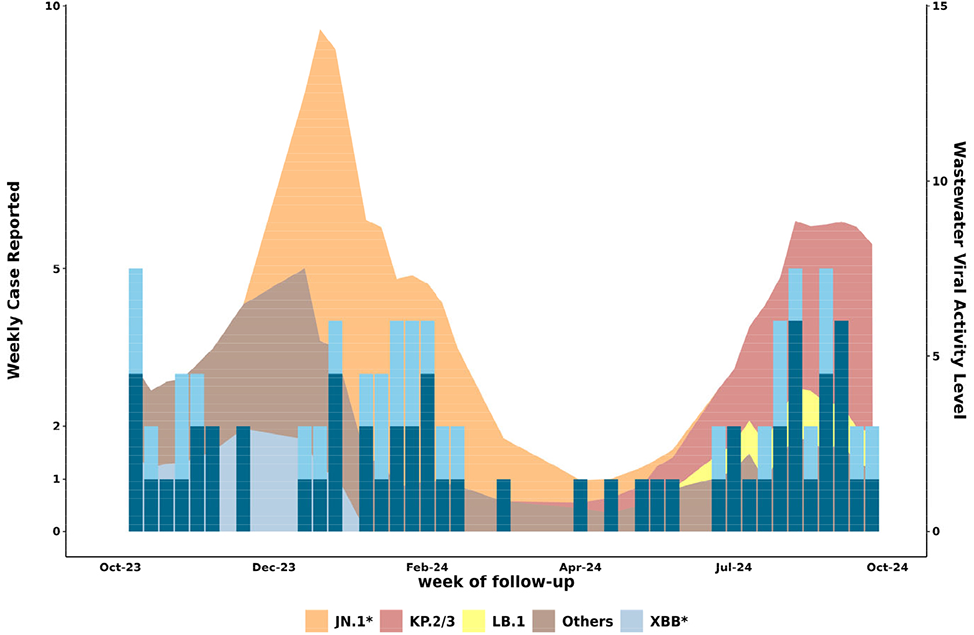
SARS-CoV-2 infection incidence in immunosuppressed participants over time, in context of variant circulation Dark and light blue bars represent number of incident COVID-19 cases in SOTR and non-SOTR immunocompromised participants, respectively, during a given calendar week (primary [right] Y axis). Background area plot represents level of community viral circulation per CDC wastewater monitoring, with colors representing relative proportions of circulating SARS-CoV-2 variants over time (secondary [left] Y axis).

**Figure:**
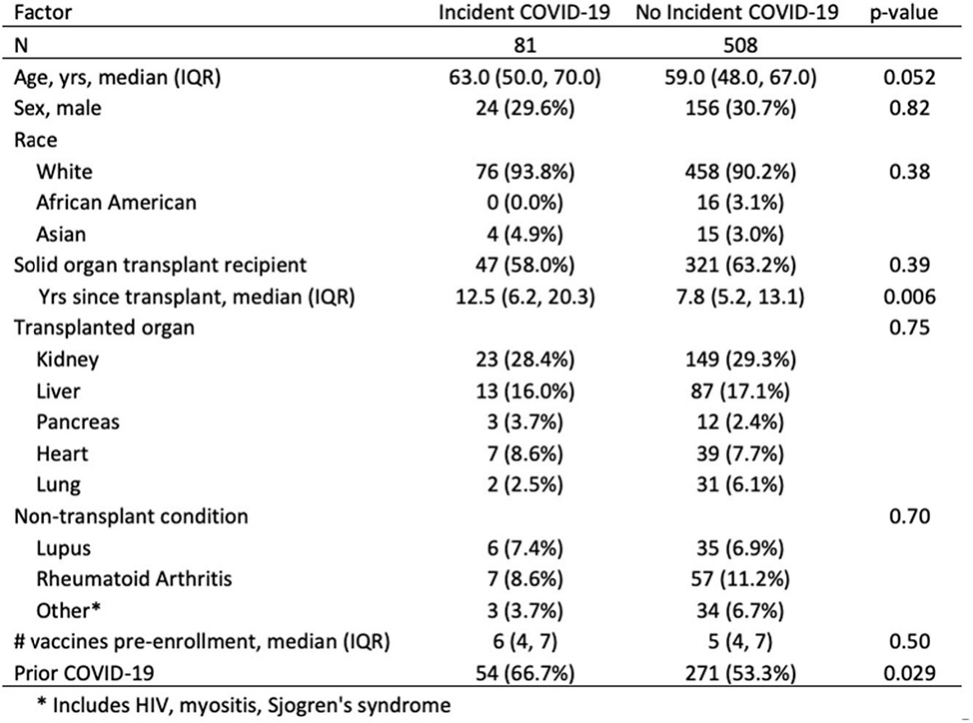
Demographics, immunocompromising condition, and vaccination history of participants with vs. without incident SARS-CoV-2 infection

**Methods:**

In a prospective national cohort, immunocompromised persons were surveyed on enrollment and 2, 4, 12, 26 weeks following any reported vaccination or infection event for incident SARS-CoV-2 infection (+antigen or PCR) from Oct. 2023-Oct. 2024; continuous unsolicited reporting was permitted. Infection incidence (# incident infections / total enrolled) was plotted by month and compared to US wastewater circulation (uL/mL) as well as CDC circulating variant proportions. Characteristics of immunocompromised persons reporting vs not reporting infections were compared. Breakthrough infection was defined as ≥14 days post XBB monovalent vaccination.

**Results:**

Among 589 immunocompromised persons (371 [63%] solid organ transplant recipients [SOTRs]), there were 81 infections with incidence rate (IR) 13.2/100 person-years (PY) (95CI 10.3-16.8). IR was similar between SOTRs and non SOTRs (12.4 vs 14.5/100 PY). IR temporally varied, with highest peaks Jan-Feb 2024 (22/100 PY) and Aug-Sept 2024 (20/100 PY), corresponding to wastewater JN.1 and KP.2/3 surges, respectively; few infections occurred in Mar-Jun 2024 (Fig). Those reporting incident infections were older, further from SOT, and had more prior infections (Table). Median days from XBB vaccine to breakthrough was 115 (59-164); 32% were within 90 days.

**Conclusion:**

In the modern SARS-CoV-2 era, infections in immunocompromised persons rise during peaks in wastewater circulation of novel variants. Clinicians may use changes in wastewater levels as a component of counseling regarding infection risk in immunocompromised patients, with relevance to risk behaviors and booster vaccination strategies.

**Disclosures:**

Andrew H. Karaba, MD PhD, GSK: Advisor/Consultant|Hologic: Advisor/Consultant William Werbel, MD PhD, AstraZeneca: Advisor/Consultant|Novavax: Advisor/Consultant

